# Supplementary material for: Metabolomic signatures of SSRI exposure during neural differentiation and correlation of lysophosphatidylcholines with early symptoms of neurodevelopmental disorders
Source: eBioMedicine. 2026 May 18;128:106291. doi: 10.1016/j.ebiom.2026.106291 (PMC13213235; doi:10.1016/j.ebiom.2026.106291)
Supplement: Supplementary Figs. S1–S4 and Table S1 [file mmc1.docx]

**Supplementary Information**

**Metabolomic signatures of SSRI exposure during neural differentiation and correlation of lysophosphatidylcholines with early symptoms of neurodevelopmental disorders**

Abishek Arora,^a,b,c^ Kristina Vacy,^d,e,i^ Cátia Marques,^f,i^ Mihai-Ovidiu Degeratu,^a,b^ Francesca Mastropasqua,^a,b^ Jenny Humphrey,^a,b^ Xuan Ye,^a,b^ Marika Oksanen,^a,b^ Peter Vuillermin,^d,g^ Anne-Louise Ponsonby,^d,g^ Ingela Lanekoff,^f,h^ and Kristiina Tammimies^a,b,*^, the Barwon Infant Study Investigator Group

^a^Center of Neurodevelopmental Disorders (KIND), Centre for Psychiatry Research, Department of Women's and Children's Health, Karolinska Institutet, and Child and Adolescent Psychiatry, Stockholm Health Care Services, Region Stockholm, Stockholm, Sweden.

^b^Astrid Lindgren Children’s Hospital, Karolinska University Hospital, Region Stockholm, Stockholm, Sweden.

^c^Science for Life Laboratory, Karolinska Institutet, Stockholm, Sweden.

^d^Florey Institute of Neuroscience and Mental Health, University of Melbourne, Victoria, Australia

^e^Melbourne School of Population and Global Health, University of Melbourne, Victoria, Australia.

^f^Department of Chemistry for Life Sciences, Uppsala University, Uppsala, Sweden.

^g^Murdoch Children's Research Institute, Royal Children's Hospital, and Department of Paediatrics, University of Melbourne, Victoria, Australia.

^h^Center of Excellence for the Chemical Mechanisms of Life, Uppsala University, Uppsala, Sweden.

^i^These authors contributed equally to this work

^*^Corresponding author. *E-mail address:* [kristiina.tammimies@ki.se](mailto:kristiina.tammimies@ki.se) (K. Tammimies)

**Supplementary Table S1. Characteristics of induced pluripotent stem cell lines included in the study.**

| **Published Name** | **Name in Study** | **RRID** | **Cell Type** | **Source Organism** | **Sex** | **Clinical Background** | **QC & Source Ref.** |
| --- | --- | --- | --- | --- | --- | --- | --- |
| AF22 | CTRL_Female_ | CVCL_A5CW | iPSC | *Homo sapiens* | Female | Neurotypical Control | Falk et al., 2012 (PLOS One) |
| CTRL-9-II | CTRL_Male_ | CVCL_JL74 | iPSC | *Homo sapiens* | Male | Neurotypical Control | Uhlin et al., 2017 (Stem Cell Research) |
| HNRNPU_del/+_ | ASD_HNRNPU_ | N/A | iPSC | *Homo sapiens* | Male | ASD with *HNRNPU* heterozygous deletion | Mastropasqua et al., 2023 (Biology Open) |
| ASD_CASK_SS_ | ASD_CASK_ | N/A | iPSC | *Homo sapiens* | Male | ASD with *CASK* splice-site variant | Becker et al., 2020 (Translational Psychiatry) |

RRID: Research Resource Identifier, N/A: Not Available, iPSC: Induced Pluripotent Stem Cell, ASD: Autism Spectrum Disorder, *HNRNPU*: Heterogeneous nuclear ribonucleoprotein U, *CASK*: Calcium/Calmodulin Dependent Serine Protein Kinase, Ref.: Reference.

**Supplementary Figure S1:** **Cellular assays for method optimisations.** **A.** Representative images of CTRL_Male_ (scale bar: 100 µM) as induced pluripotent stem cells (iPSCs), neuroepithelial stem (NES) cells, NES cells 5 days post induction of neural differentiation (D5) and NES cells 28 days post induction of neural differentiation (D28). **B.** Cell viability at 24 hours (n=3 per condition) and 120 hours (day 5, n=3 per condition) of fluoxetine (FH; from Arora et al., 2023, Scientific Reports), citalopram (CH), sertraline (SH) and paroxetine (PH) exposure using the MTS assay. **C.** Optimisation of the shift from glycolysis to oxidative phosphorylation using glucose free, galactose supplemented medium and testing with sodium azide exposure using a mitotoxicity assay (cell membrane permeability, n=3 per condition and total ATP, n=3 per condition). **D.** Detection of FH, CH, SH, and PH in the mass spectrometry readout following *in-vitro* exposure at day 5 (n=24 per condition) and 28 (n=24 per condition).

**Supplementary Figure S2:** **Analysis of selective serotonin reuptake inhibitor (SSRI) exposure effects.** **A.** Principal component analysis (PCA) scatter plot of detected metabolites following fluoxetine (FH), citalopram (CH), sertraline (SH) and paroxetine (PH) exposure at day 5 (n=24 per condition). **B.** PCA scatter plot of detected metabolites following FH, CH, SH and PH exposure at day 28 (n=24 per condition). **C.** Interaction network of top 50 enriched metabolic pathways following SH exposure at day 5. **D.** Interaction network of top 50 enriched metabolic pathways following PH exposure at day 5. **E.** *In-vitro* assay determined total ROS levels at day 5 (Control, n=6; lysophosphatidylcholines, LPC, n=8; AACOCF_3_, n= 6; LPC+AACOCF_3_, n=8) and day 28 (Control, n=7; LPC, n=8; AACOCF_3_, n= 4; LPC+AACOCF_3_, n=4) of LPC inhibition (two-way ANOVA, Tukey’s post hoc adjusted p: *<0.05, **<0.01, ***<0.001). **F.** *In-vitro* assay determined total ATP levels at day 5 (Control, n=8; LPC, n=8; AACOCF_3_, n= 6; LPC+AACOCF_3_, n=8) and day 28 (Control, n=8; LPC, n=8; AACOCF_3_, n= 4; LPC+AACOCF_3_, n=4) of LPC inhibition (two-way ANOVA, Tukey’s post hoc adjusted p *<0.05, **<0.01, ***<0.001).

**Supplementary Figure S3:** **Univariate analysis of selective serotonin reuptake inhibitor (SSRI) exposure effects.** **A.** Volcano plots for differentially detected metabolites following SSRI exposure at day 5. **B.** Volcano plots for differentially detected metabolites following SSRI exposure at day 28.

**Supplementary Figure S4:** **Selective serotonin reuptake inhibitor (SSRI) effects as per clinical diagnostic group.** **A.** *In-vitro* assay determined total ROS levels at day 5 (n=12 per condition) and day 28 (n=12 per condition) of fluoxetine (FH), citalopram (CH), sertraline (SH) and paroxetine (PH) exposure in the non-ASD and ASD lines (Kruskal-Wallis Rank Sum, Nemenyi post hoc p: *<0.05, **<0.01, ***<0.001). **B.** *In-vitro* assay determined total ATP levels at day 5 (n=12 per condition; two-way ANOVA, Tukey’s post hoc adjusted p: *<0.05, **<0.01, ***<0.001) and day 28 (n=12 per condition; Dunn (1964) Kruskal-Wallis, Holm post hoc p: *<0.05, **<0.01, ***<0.001) of FH, CH, SH and PH exposure in the non-ASD and ASD lines. **C.** Heatmap of estimate values from the applied mixed linear model for metabolites and SSRIs, with a significant overlap across at least two SSRIs in the non-ASD lines at Day 5 (FDR adjusted p: a<0.05, b<0.01, c<0.001). **D.** Heatmap of estimate values from the applied mixed linear model for metabolites and SSRIs, with a significant overlap across at least two SSRIs in the ASD lines at Day 5 (FDR adjusted p: a<0.05, b<0.01, c<0.001). **E.** Heatmap of estimate values from the applied mixed linear model for metabolites and SSRIs, with a significant overlap across at least two SSRIs in the non-ASD lines at Day 28 (FDR adjusted p: a<0.05, b<0.01, c<0.001). **F.** Heatmap of estimate values from the applied mixed linear model for metabolites and SSRIs, with a significant overlap across at least two SSRIs in the ASD lines at Day 28 (FDR adjusted p: a<0.05, b<0.01, c<0.001).
