# Supplementary material for: Metabolomic signatures of SSRI exposure during neural differentiation and correlation of lysophosphatidylcholines with early symptoms of neurodevelopmental disorders
Source: eBioMedicine. 2026 May 18;128:106291. doi: 10.1016/j.ebiom.2026.106291 (PMC13213235; doi:10.1016/j.ebiom.2026.106291)
Supplement: Study_Group_Members [file mmc7.docx]

**Study Group Members**

**Metabolomic signatures of SSRI exposure during neural differentiation and correlation of lysophosphatidylcholines with early symptoms of neurodevelopmental disorders**

| **First Name** | **Last Name** |
| --- | --- |
| David | Burgner |
| Erica | Grundell |
| Katherine | Drummond |
| Len | Harrison |
| Luba | Sominsky |
| Martin | O’Hely |
| Mimi | Tang |
| Peter | Sly |
| Richard | Saffery |
| Samantha | Dawson |
| Toby | Mansell |
